# Simple One‐Step Molten Salt Method for Synthesizing Highly Efficient MXene‐Supported Pt Nanoalloy Electrocatalysts

**DOI:** 10.1002/advs.202303693

**Published:** 2023-10-20

**Authors:** Ya Wang, Lili Li, Miao Shen, Rui Tang, Jing Zhou, Ling Han, Xiuqing Zhang, Linjuan Zhang, Guntae Kim, Jian‐Qiang Wang

**Affiliations:** ^1^ Shanghai Institute of Applied Physics Chinese Academy of Sciences Shanghai 201800 China; ^2^ University of Chinese Academy of Sciences Beijing 100049 China; ^3^ State Key Laboratory of Crystal Materials and Institute of Crystal Materials Shandong University Jinan 250100 China; ^4^ School of Mechanical and Power Engineering East China University of Science and Technology 200237 Shanghai China

**Keywords:** catalysts, molten salts, MXenes, platinum nanoalloys

## Abstract

MXene‐supported noble metal alloy catalysts exhibit remarkable electrocatalytic activity in various applications. However, there is no facile one‐step method for synthesizing these catalysts, because the synthesis of MXenes requires a strongly oxidizing environment and the preparation of platinum nanoalloys requires a strongly reducing environment and high temperatures. Hence, achieving coupling in one step is extremely challenging. In this paper, a straightforward one‐step molten salt method for preparing MXene‐supported platinum nanoalloy catalysts is proposed. The molten salt acts as the reaction medium to dissolve the transition metals and platinum ions at high temperatures. Transition metal ions oxidize the A‐site element from its MAX precursor at high temperatures, and the resulting transition metals further reduce platinum ions to form alloys. By coupling Al oxidation and platinum ion reduction using a molten salt solvent, this method directly converts Ti_3_AlC_2_ to a Pt‐M@Ti_3_C_2_T*_x_* catalyst (where M denotes the transition metal). It further offers the possibility of extending the Pt‐M phase to binary, ternary, or quaternary platinum‐containing nanoalloys and converting the Al‐containing MAX phase to Ti_2_AlC and Ti_3_AlCN. Due to the strong interfacial interaction, the as‐prepared Pt‐Co@Ti_3_C_2_T*_x_* is superior to commercial Pt/C (20 wt.%) in the hydrogen evolution reaction.

## Graphical abstract

This method enables the direct conversion of Ti_3_AlC_2_ into a Pt‐M@Ti_3_C_2_T*_x_* (where M denotes the transition metal) in one step, with the possibility of extending the Pt‐M phase to binary, ternary, or quaternary platinum‐containing alloys and converting the Al‐containing MAX phase into Ti_2_AlC and Ti_3_AlCN. Due to the strong interfacial interaction, as‐prepared Pt‐Co@Ti_3_C_2_T*_x_* exhibits high performance in the hydrogen evolution reaction.

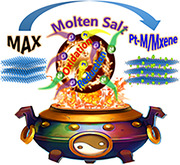

## 1 Introduction

Although platinum is a highly efficient catalyst, its widespread use is hindered by its high cost and limited availability.^[^ ^1^, ^2^, ^3^, ^4^ ^]^ One effective strategy to overcome these challenges is to form nanoalloys by doping platinum with non‐noble metals and dispersing these nanoalloys onto a suitable carrier. This approach enhances the number of active sites, reduces the cost of working with noble metal catalysts, and improves their utilization efficiency.^[^ ^5^, ^6^, ^7^, ^8^, ^9^ ^]^

MXenes are a family of 2D compounds consisting of transition metal carbides and nitrides.^[^ ^10^, ^11^, ^12^ ^]^ Owing to their adjustable band gap, high specific surface area, good electronic conductivity, and mechanical stability, MXenes are commonly used as carriers for loading noble metals and alloys, exhibiting excellent catalytic activity in many electrocatalytic reactions such as the hydrogen evolution reaction (HER),^[^ ^13^, ^14^, ^15^ ^]^ oxygen evolution reaction (OER),^[^ ^16^ ^]^ oxygen reduction reaction (ORR),^[^ ^17^, ^18^ ^]^ and methanol oxidation reaction.^[^ ^19^ ^]^ The preparation of MXene‐supported noble metal alloy catalysts consists of three main steps: preparation of the MXene carrier, adsorption and subsequent reduction of platinum and other metal ions on MXenes, and the formation of a platinum alloy. This process leads to complications during the synthesis of MXene‐based noble metal alloy catalysts, requiring different chemical environments for each step.^[^ ^13^, ^20^, ^21^, ^22^ ^]^ For example, the preparation of MXenes requires a strong oxidizing‐solvent environment to remove the A‐site element from its MAX precursor. In contrast, the adsorption of metal ions requires a mild solvent with high platinum solubility and other metal ions that do not destroy the structure of MXenes. The reduction and alloying processes require strong reducing agents and high temperatures. The main challenge in simplifying the preparation of MXene‐supported Pt‐based nanoalloy catalysts is combining the different chemical environments of oxidation, reduction, mild solvents, and high temperatures in one process.

In recent years, the synthesis of MXenes by Lewis acid etching of MAX with a high‐temperature molten salt as a solvent has been developed.^[^ ^11^, ^12^, ^23^ ^]^ By tuning the concentration and type of metallic ions in the molten salt, the oxidation environment can be adjusted to etch the A‐site element in MAX. This process allows for the generated MXenes to stably exist in mild, high‐temperature molten salts and is accompanied by the production of a reducing metal. Our research was inspired by the coupled environment of oxidation, reduction, and metal ion dissolution in high‐temperature molten salts during the preparation of MXenes. We developed a one‐step method to prepare MXene‐supported platinum nanoalloy catalysts by coupling Al oxidation and platinum ion reduction using a molten salt solution containing transition metals and platinum chlorides. This method enables the direct conversion of Ti_3_AlC_2_ into a Pt‐M@Ti_3_C_2_T*_x_* catalyst (where M denotes the transition metal), with the possibility of extending the Pt‐M phase to binary, ternary, or quaternary platinum‐containing alloys and converting the Al‐containing MAX phase into Ti_2_AlC and Ti_3_AlCN. Our results show that Pt‐Co@Ti_3_C_2_T*_x_* exhibits superior electrocatalytic performance in the HER compared to commercial Pt/C (20 wt.%) because of an interfacial charge redistribution between the Pt‐Co nanoalloys and the Ti_3_C_2_T*_x_* substrate.

## 2 Results and Discussion

### 2.1 Characterization of the As‐Synthesized Materials

**Figure 1:**
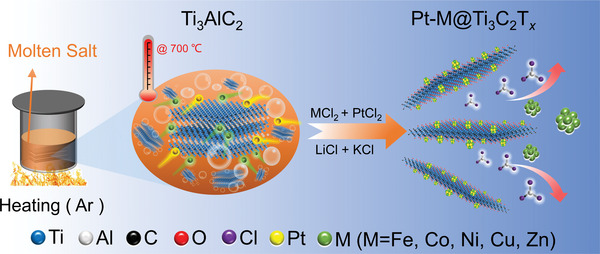
Schematic illustration of the formation of Pt‐M@MXene.

**Figure** **1** illustrates the synthesis of Pt‐M@MXene. The Al‐containing compounds Ti_3_AlC_2_, Ti_2_AlC, and Ti_3_AlCN were used as precursors for the preparation of MXenes. The etching agents used for the synthetic process included CoCl_2 ∙6H2O_, NiCl_2_, FeCl_2_, ZnCl_2_, and CuCl_2_, while PtCl_2_ was used as the noble metal reaction agent. Herein, we utilized Ti_3_AlC_2_, CoCl_2∙6H2O ,_ and PtCl_2_ as reaction models to prepare 25Pt‐Co@Ti_3_C_2_T*_x_*. The details of the synthesis are summarized in the experimental section and in Table S1 (Supporting Information).

**Figure 2:**
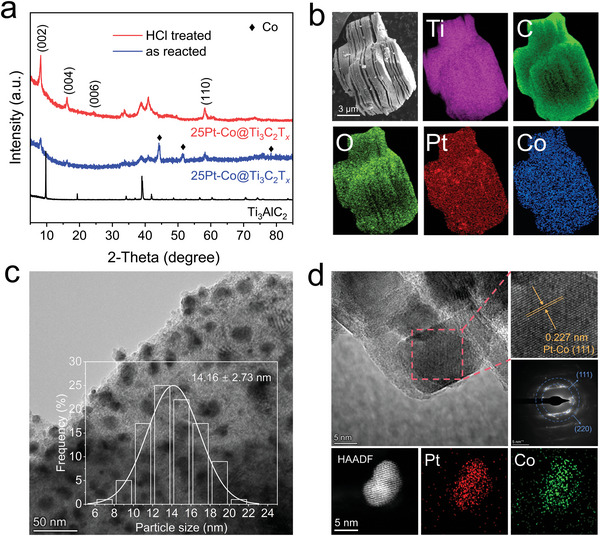
Structural characterization of 25Pt‐Co@Ti_3_C_2_T*_x_*. a) XRD patterns of the Ti_3_AlC_2_ and 25Pt‐Co@Ti_3_C_2_T*_x_* products. b) SEM image and the related EDS mapping of the Pt, Co, O, Ti, and C elements for 25Pt‐Co@Ti_3_C_2_T*_x_*. c) TEM image and size distribution of Pt‐Co nanoparticles. d) HRTEM image, SAED pattern and HAADF‐STEM image of 25Pt‐Co@Ti_3_C_2_T*_x_*.

X‐ray diffraction (XRD) results (**Figure** **2**a) reveal that the Ti_3_AlC_2_ samples react with CoCl_2_∙6H2O and PtCl_2_ in the molten salt, resulting in a shift of the (002) peak from 9.86° to 8.11°, indicating a conversion from pristine Ti_3_AlC_2_ to 25Pt‐Co@Ti_3_C_2_T*_x_*.^[^ ^24^, ^25^ ^]^ Washing the samples with 2 m hydrochloric acid eliminates the characteristic peaks of the by‐product Co at 44.13°, 51.42°, and 75.80°,^[^ ^12^ ^]^ similar to the behavior observed in Ti_3_C_2_T*_x_* samples etched with only CoCl_2∙6H2O_ (Figure S1a, Supporting Information). However, for 100Pt‐Co@Ti_3_C_2_T*_x_*, the addition of more PtCl_2_ results in peaks at 41.2°, 47.9°, 69.9°, and 84.7° (Figure S1b, Supporting Information). These peaks are slightly different from the (111), (200), (220), and (311) peaks of pure Pt and of Pt@Ti_3_C_2_T*_x_* etched with only PtCl_2_ (Figure S1c, Supporting Information), which can be attributed to the formation of a Pt‐Co alloy owing to the introduction of Co into the Pt.^[^ ^5^, ^13^, ^26^ ^]^ The 25Pt‐Co@Ti_3_C_2_T*_x_* sample exhibits an accordion‐like layered structure, as observed using scanning electron microscopy (SEM) (Figure 2b).^[^ ^27^, ^28^ ^]^ Both Pt and Co are evenly distributed on the Ti_3_C_2_T*_x_* surface. Scanning transmission electron microscopy (STEM) reveals the presence of dispersed nanoparticles with a mean size of ≈14 nm on the surface (Figure 2c). Upon the addition of PtCl_2_ or decreasing of temperature, the average size of the nanoparticles increases (Figures S2 and S3, Supporting Information). Furthermore, a high‐resolution TEM image (Figure 2d) confirms the presence of a Pt‐Co alloy with a lattice spacing of 0.227 nm, which is between those of pure Co (0.217 nm) and Pt (111) (0.230 nm).^[^ ^8^, ^29^ ^]^ The selected‐area electron diffraction (SAED) pattern shows a regular hexagonal lattice (Figure S4, Supporting Information), which is in good agreement with the lattice pattern of Ti_3_C_2_T*_x_*. The smaller region SAED in Figure 2d displays diffraction rings, which are assigned from the inside to the outside, corresponding to the (111) and (220) crystal planes of the Pt‐Co alloy.^[^ ^30^ ^]^ High‐angle annular dark field scanning transmission electron microscopy (HAADF‐STEM) mapping further confirms that the dispersed nanoparticles on Ti_3_C_2_T*_x_* are composed of Pt and Co (Figure 2d; Figure S5, Supporting Information). The contents of Pt and Co are 4.68 and 1.58 wt.%, as determined by inductively coupled plasma optical emission spectroscopy (ICP‐OES, Table S2, Supporting Information).

By replacing CoCl_2_∙6H2O with NiCl_2_, FeCl_2_, or CuCl_2_ as an etching agent, other platinum‐based alloys such as 25Pt‐Ni@Ti_3_C_2_T*_x_* (Figure S6 and Table S3, Supporting Information), 25Pt‐Fe@Ti_3_C_2_T*_x_* (Figure S7 and Table S3, Supporting Information), and 25Pt‐Cu@Ti_3_C_2_T*_x_* (Figure S8, Supporting Information) have been produced. Moreover, simultaneously using two or three etching agents enables the preparation of ternary or quaternary Pt alloys, such as 25Pt‐Ni‐Co@Ti_3_C_2_T*_x_* (Figure S9, Supporting Information) and 25Pt‐Fe‐Co‐Ni@Ti_3_C_2_T*_x_* (Figure S10, Supporting Information) on Ti_3_C_2_T*_x_*. Al‐containing MAX can be extended to Ti_2_AlC and Ti_3_AlCN to produce 25Pt‐Co@Ti_3_CNT*_x_* (Figure S11, Supporting Information) and 25Pt‐Co@Ti_2_CT*_x_* catalysts (Figure S12, Supporting Information).

**Figure 3:**
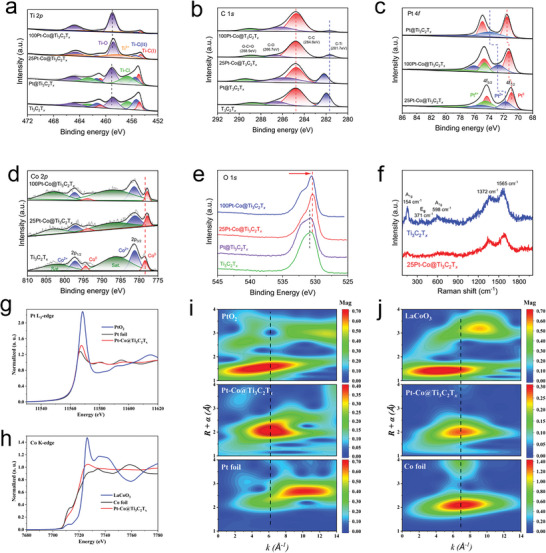
Spectroscopic characterization of 25Pt‐Co@Ti_3_C_2_T*_x_*. a) Ti 2p and b) C 1s XP spectra of Ti_3_C_2_T*_x_*, Pt@Ti_3_C_2_T*_x_*, 25Pt‐Co@Ti_3_C_2_T*_x_* and 100Pt‐Co@Ti_3_C_2_T*_x_*. c) Pt 4f XP and d) Co 2p spectra of Pt@Ti_3_C_2_T*_x_*, 25Pt‐Co@Ti_3_C_2_T*_x_* and 100Pt‐Co@Ti_3_C_2_T*_x_*. e) O 1s XP spectra of Ti_3_C_2_T*_x_*, Pt@Ti_3_C_2_T*_x_*, 25Pt‐Co@Ti_3_C_2_T*_x_* and 100Pt‐Co@Ti_3_C_2_T*_x_*. f) Raman spectra of Ti_3_C_2_T*_x_* and 25Pt‐Co@Ti_3_C_2_T*_x_*. g) Normalized X‐ray absorption near‐edge structure (XANES) spectra of the Pt L_3_‐edge for 25Pt‐Co@Ti_3_C_2_T*_x_*, PtO_2_, and Pt foil. h) Normalized XANES spectra of the Co K‐edge for 25Pt‐Co@Ti_3_C_2_T*_x_*, LaCoO_3_, and Co foil. i) Wavelet transform for the k^2^‐weighted extended X‐ray absorption fine structure (EXAFS) spectra of 25Pt‐Co@Ti_3_C_2_T*_x_*, PtO_2_, and Pt foil. j) Wavelet transform for the k^2^‐weighted EXAFS spectra of 25Pt‐Co@Ti_3_C_2_T*_x_*, LaCoO_3_, and Co foil.

To gain further insight into the surface structure of 25Pt‐Co@Ti_3_C_2_T*_x_*, several techniques, including X‐ray photoelectron spectroscopy (XPS), Raman spectroscopy, XANES, and EXAFS were employed. In the high‐resolution Ti 2p spectrum (**Figure** **3**a; Figure S13, Supporting Information), the peaks at 454.63 and 455.47 eV correspond to the Ti‐C (I) (2p_3/2_) and Ti‐C (II) (2p_3/2_) bonds, respectively.^[^ ^31^ ^]^ These results suggest a reduction of these bonds from 12.72% and 20.52% in the Ti_3_C_2_T*_x_* percentage to 8.45% and 12.07% in the 25Pt‐Co@Ti_3_C_2_T*_x_* percentage (Table S4, Supporting Information). Furthermore, the Ti 2p spectrum reveals that upon the addition of platinum to Ti_3_C_2_T*_x_*, the peak corresponding to the Ti‐O (2p_3/2_) bond increases from 25.71% to 44.17% in 25Pt‐Co@Ti_3_C_2_T*_x_* and 69.3% in 100Pt‐Co@Ti_3_C_2_T*_x_* (Table S4, Supporting Information). In contrast, the peak corresponding to the Ti‐Cl (2p_3/2_) bond decreases from 41.05% to 35.31% and 23.01%, respectively (Table S4, Supporting Information).^[^ ^32^ ^]^ These findings suggest a transformation of the functional groups, whereby ‐Cl is substituted with ‐O after incorporating Pt‐Co alloy into Ti_3_C_2_T*_x_*. The C 1s spectrum of the 25Pt‐Co@Ti_3_C_2_T*_x_* sample (Figure 3b; Table S5, Supporting Information) exhibits a much weaker Ti‐C bond signal compared to Ti_3_C_2_T*_x_* and Pt@Ti_3_C_2_T*_x_*. The high‐resolution Pt 4f spectrum of 25Pt‐Co@Ti_3_C_2_T*_x_* (Figure 3c) shows three peaks at 70.93/74.33, 71.72/75.12, and 74.50/77.90 eV, which can be attributed to Pt^0^, Pt^2+^, and Pt^4+^, respectively.^[^ ^2^, ^33^, ^34^ ^]^ Compared with the high‐resolution Pt 4f spectrum of Pt nanoparticles and Pt@Ti_3_C_2_T*_x_* (Figure S14, Supporting Information), the formation of Pt‐Co alloy on Ti_3_C_2_T*_x_* will cause the Pt to shift to a lower valence state.^[^ ^35^ ^]^ Similarly, the Co 2p spectrum in Figure 3d confirms the presence of Co^2+^ (2p_3/2_, 781.25 eV) and Co^0^ (2p_1/2_778.50 eV).^[^ ^5^, ^36^ ^]^ Notably, the introduction of Pt‐Co nanoalloy causes a negative shift by 0.64 eV in the binding energy of Co 2p. However, with the change of the atom ratio of Pt:Co, the binding energy of Co 2p does not change significantly (Figure S15, Supporting Information). Therefore, the formation of Pt‐Co nanoalloys on Ti_3_C_2_T*_x_* substrate results in the shift of both Pt 4f and Co 2p toward lower binding energy. In addition, compared to Ti_3_C_2_T*_x_* and Pt@Ti_3_C_2_T*_x_*, the O 1s XPS peak of Pt‐Co@Ti_3_C_2_T*_x_* is negatively shifted by 0.45 eV (Figure 3e).^[^ ^37^ ^]^ These results indicate an interaction between Pt‐Co and the electron‐withdrawing ‐O species on Ti_3_C_2_T*_x_*, causing electron transfer from the Ti_3_C_2_T*_x_* substrate to the Pt and Co atoms. Furthermore, the Ti 2p spectra exhibit a significant increase in the Ti‐O bond (Figure S13a and Table S4, Supporting Information) and a marked decrease in the Ti‐C bond (Figure S13b and Table S5, Supporting Information) as the Pt content in Pt‐Co@Ti_3_C_2_T*_x_* increases.^[38]^ The O/Ti ratio calculated from the XPS data shows a positive correlation with the Pt content (Figure S16a, Supporting Information), consistent with the EDS data (Figure S16b, Supporting Information). These results further suggest that the Pt‐Co nanoparticles on the Ti_3_C_2_T*_x_* surface are likely immobilized via Ti‐O bonds. The Raman spectrum of Ti_3_C_2_T*_x_* (Figure 3f) displays strong A_1g_ signals at 154 cm^−1^, corresponding to the A_1g_ out‐of‐plane vibrational mode of the C and Ti atoms. The A_1g_ mode of 25Pt‐Co@Ti_3_C_2_T*_x_* weakens, indicating that the vibration modes of the C and Ti atoms in Ti_3_C_2_T*_x_* are affected by Pt‐Co loading. Ti_3_C_2_T*_x_* shows a relatively weak E_g_ signal at 371 cm^−1^ and an A_1g_ signal at 598 cm^−1^, corresponding to the vibrational modes of the C and Ti atoms and terminal groups.^[^ ^21^, ^33^, ^38^ ^]^ Owing to Pt‐Co bonding with the surface group of 25Pt‐Co@Ti_3_C_2_T*_x_*, the E_g_ peak at 371 cm^−1^ disappears, and the A_1g_ peak at 598 cm^−1^ shifts. The accurate local electronic structures and atomic coordination of Pt and Co were studied using X‐ray absorption spectra (XAS). Figure 3g shows the XANES spectra at the Pt L_3_‐edge of 25Pt‐Co@Ti_3_C_2_T*_x_* with Pt^0^ (Pt foil) and Pt^4+^ (PtO_2_), indicating that the valence state of Pt in 25Pt‐Co@Ti_3_C_2_T*_x_* resides between the Pt foil and PtO_2_; this is consistent with the XPS results. The wavelet transform (WT) of the EXAFS spectrum for Pt is shown in Figure 3i. The maximum WT intensity of Pt in 25Pt‐Co@Ti_3_C_2_T*_x_* is located at 2.1 Å in R‐space and 6.1 Å^−1^ in k‐space. These results differ from those of Pt foil (2.7 Å in R‐space and 10 Å^−1^ in k‐space) and PtO_2_ (1.5 Å in R‐space and 5 Å^−1^ in k‐space), reflecting the strong metal‐support interaction between Pt and the Ti_3_C_2_T*_x_* substrate.^[^ ^5^ ^]^ The absorption edge energy at the Co K‐edge of 25Pt‐Co@Ti_3_C_2_T*_x_* is close to that of the Co foil (Figure 3h), indicating the presence of Co^0^. It is worth noting that the XANES shape of Co in 25Pt‐Co@Ti_3_C_2_T*_x_* is different from that of the Co foil, which is attributed to the difference in structural symmetry between the Pt‐Co alloys and Co foil. As shown in Figure 3j, the maximum WT intensity of Co nearly overlaps with that of the Co foil, corresponding to a small shift in the R‐space. This shift may be caused by the formation of Pt‐Co alloy nanoparticles. Based on a comprehensive analysis of the surface structures of 25Pt‐Co@Ti_3_C_2_T*_x_*, it is speculated that a strong interaction between the Pt‐Co nanoparticles and the Ti_3_C_2_T*_x_* substrate benefits its electrocatalytic activity_._

### 2.2 Electrocatalytic Properties for the HER

**Figure 4:**
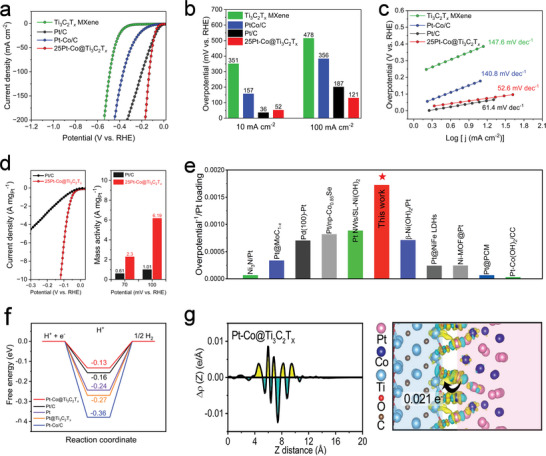
Electrocatalytic performance of 25Pt‐Co@Ti_3_C_2_T*_x_*. a) Polarization curves of Ti_3_C_2_T*_x_*, 25Pt‐Co@Ti_3_C_2_T*_x_*, Pt‐Co/C, and commercial Pt/C (20 wt.%). b) Specific activity of electrocatalysts at 10 mA cm^−2^ and 100 mA cm^−2^. c) Tafel plots measured at 10 mA cm^−2^. d) Specific mass activities at 100 and 200 mV. e) Property comparison of 25Pt‐Co@Ti_3_C_2_T*_x_* with reported Pt‐based catalysts, where the axis of ordinates represents the reciprocal of the overpotential of the catalyst at 10 mA cm^−2^ divided by the platinum loading. f) Free energy diagram for the HER on Pt (111), Pt/C, Pt@Ti_3_C_2_T*_x_*, Pt‐Co/C, and Pt‐Co@Ti_3_C_2_T*_x_*. g) Planar average electron density difference (Δρ) is integrated over the x‐y plane for Pt‐Co@Ti_3_C_2_T*_x_* as a function of the z distance. The shadow is the charge density difference on the interface. The yellow and cyan colors indicate electron accumulation and depletion, respectively. The arrow shows the direction of the electron transfer.

As previously discussed, the interface effect between the Pt‐Co alloy and the Ti_3_C_2_T*_x_* carrier is likely to optimize the electronic structure, leading to high efficiency and excellent electrocatalytic performance. Therefore, linear sweep voltammetry (LSV) was performed on the 25Pt‐Co@Ti_3_C_2_T*_x_* catalyst in 1.0 m KOH (**Figure** **4**a; Figure S17a, Supporting Information) to evaluate its HER activities. As shown in Figure 4b and Figure S17b (Supporting Information), the activity of Pt‐Co@Ti_3_C_2_T*_x_* is related to the proportion of Pt atoms in the samples, among which 25Pt‐Co@Ti_3_C_2_T*_x_* exhibited optimal activity, with the overpotential of 52 mV at 10 mA cm^−2^, much lower than those of Ti_3_C_2_T*_x_* (351 mV) and Pt‐Co/C (157 mV). However, it is slightly higher than that of commercial 20 wt.% Pt/C (36 mV). At current densities above 30 mA cm^−2^, 25Pt‐Co@Ti_3_C_2_T*_x_* exhibits superior performance with an overpotential of 121 mV at 100 mA cm^−2^, outperforming Pt/C, which exhibits an overpotential of 187 mV. Figure 4c shows the calculated Tafel slope, which is typically used to assess the reaction kinetics of catalysts. The slope of 25Pt‐Co@Ti_3_C_2_T*_x_* (52.6 mV dec^−1^) is lower than those of Pt/C (61.4 mV dec^−1^), Pt‐Co/C (140.8 mV dec^−1^), and Ti_3_C_2_T*_x_* (147.6 mV dec^−1^), implying faster kinetics of the HER. Electrochemical impedance spectroscopy (EIS) was performed to further investigate the HER kinetics (Figure S18 and Table S6, Supporting Information). The 25Pt‐Co@Ti_3_C_2_T*_x_* exhibits minimal charge‐transfer resistance of 10.9 Ω, and significantly less than those of Pt/C (18.6 Ω), Pt‐Co/C (41.5 Ω) and Ti_3_C_2_T*_x_* (45.2 Ω), indicating that a strong covalent interaction between Pt‐Co nanoparticles and the Ti_3_C_2_T*_x_* MXene substrates accelerates electron/ion migration. To quantitatively compare the electrochemical activities of 25Pt‐Co@Ti_3_C_2_T*_x_* and commercial Pt/C, their mass activities were determined at various potentials (Figure 4d). The mass activity of 25Pt‐Co@Ti_3_C_2_T*_x_* reaches 6.19 A mg_Pt_^−1^ at −100 mV, five times higher than that of commercial 20 wt.% Pt/C (1.01 A mg_Pt_^−1^) in an alkaline medium. The electrochemically active surface area (ECSA) of samples was investigated by using double‐layer capacitance (Cdl) to estimate the electrocatalytic activity (Figure S19, Supporting Information). The slope for the 25Pt‐Co@Ti_3_C_2_T*_x_* electrode is 5.13 mF cm^−2^, which is larger than that of commercial Pt/C (4.29 mF cm^−2^), indicating that the ECSA for 25Pt‐Co@Ti_3_C_2_T*_x_* in alkaline solution is improved. The ECSA normalized hydrogen evolution current (j) was used to highlight the intrinsic catalytic activity (Figure S20, Supporting Information). In addition to the aforementioned advantages, the 25Pt‐Co@Ti_3_C_2_T*_x_* electrode demonstrates excellent stability compared to commercial 20 wt.% Pt/C, showing no significant decrease in performance after 25 h. In particular, the performance of 25Pt‐Co@Ti_3_C_2_T*_x_* is superior to that of Pt/C at a high current density of 120 mA cm^−2^ (Figure S21, Supporting Information). This exceptional performance also exceeds that of many recently reported noble metal catalysts with an average size greater than 2 nm (Figure 4e; Table S7, Supporting Information).

To understand the excellent HER electrocatalytic activity of Pt‐Co@Ti_3_C_2_T*_x_*, we employed DFT to investigate the hydrogen adsorption‐free energy (ΔG_H_), the dominant factor governing HER activity.^[^ ^39^ ^]^ Slab models of Pt/C, Pt@Ti_3_C_2_T*_x_*, Pt‐Co/C, and Pt‐Co@Ti_3_C_2_T*_x_* were constructed following the literature to investigate the interface effect.^[^ ^13^, ^40^ ^]^ The order of HER performance is Pt‐Co@Ti_3_C_2_T*_x_* >Pt/C>Pt@Ti_3_C_2_T*_x_* >Pt‐Co/C (Figure 4f), indicating that the Pt and Pt‐Co alloys have different degrees of coupling with the support materials. Compared to commercial Pt/C, the ΔG_H_ of Pt‐Co@Ti_3_C_2_T*_x_* is closer to the thermo‐neutral state, indicating favorable HER activity. The charge‐difference plots (Figure 4g; Figure S22, Supporting Information) indicate the coupling effect between the Pt‐Co alloy and the ‐O‐terminated Ti_3_C_2_T*_x_* substrate is the largest (≈0.021 electrons per unit cell), while the interfacial distance between Pt‐Co alloy and Ti_3_C_2_T*_x_* substrate is the smallest (about 1.84 Å). Therefore, the coupling between the Pt‐Co alloy and the substrate through interfacial charge redistribution can synergistically accelerate and optimize the HER activity, enhancing the HER toward an energetically more favorable pathway.

## 3 Conclusion

In summary, we describe a simple one‐step method to produce platinum nanoalloy catalysts supported on MXenes by reacting CoCl_2_∙6H2O and PtCl_2_ with Ti_3_AlC_2_ in molten salt. This method enables the direct conversion of Ti_3_AlC_2_ into a Pt‐Co@Ti_3_C_2_T*_x_* catalyst in one step, wherein Pt‐Co nanoparticles are uniformly dispersed on the surface of Ti_3_C_2_T*_x_*. The strong interaction between the Pt‐Co nanoparticles and the Ti_3_C_2_T*_x_* substrate benefits its electrocatalytic activity, so Pt‐Co@Ti_3_C_2_T*_x_* is superior to commercial Pt/C(20 wt.%) in HERs. Furthermore, the MAX phase concept can be applied to other MAX phases containing aluminum, such as Ti_2_AlC and Ti_3_AlCN. Additionally, Pt‐M@MXene catalysts can be expanded to include binary, ternary, or quaternary Pt‐based alloys by modifying the transition metal chloride added to the mixture. Further control of charge distribution at the interface can be achieved by introducing more than two metals into the Pt alloy. Therefore, the effects of this modified structure on other electrocatalytic performance parameters warrant further investigation. This study offers a potential avenue for developing highly efficient noble metal alloy catalysts supported by MXenes for electrocatalytic applications.

## 4 Experimental Section

### Synthesis of Pt‐Co@Ti_3_C_2_T_x_

First, 0.2 g of Ti_3_AlC_2_ powder (400 mesh, Yiyi Technology, China), 0.49 g of CoCl_2_∙6H_2_O (99.0 wt.% purity, Sinopharm Chemical Reagent Co., Ltd., China), and 0.24 g of LiCl‐KCl salt (97 wt.% purity, Sinopharm Chemical Reagent Co., Ltd., China) with a molar ratio of 1:1 were added to an agate mortar and ground thoroughly. Subsequently, 25 mg of PtCl_2_ (99.4 wt.% purity, Sinopharm Chemical Reagent Co., Ltd., China) was added to the mixture and ground. The obtained powder was transferred into a boat‐shaped alumina crucible, heated to 700 °C at a rate of 3–4 °C min^−1^, and maintained at 700 °C for 24 h in an argon‐protected glove box. After cooling to room temperature, the powders were washed with deionized (DI) water to remove the residual molten salt, followed by washing with 2 m hydrochloric acid (HCl) to remove the Co particles. The resulting solution was washed with DI water several times and dried under vacuum at 80 °C for 18 h. The obtained sample was labeled 25Pt‐Co@Ti_3_C_2_T*_x_*. The obtained samples with PtCl_2_ concentrations of 5, 15, and 100 mg were denoted as 5Pt‐Co@Ti_3_C_2_T*_x_*, 15Pt‐Co@Ti_3_C_2_T*_x_*, and 100Pt‐Co@Ti_3_C_2_T*_x_*, respectively.

### Synthesis of Other Pt‐M@MXene

The synthesis of other Pt‐M@MXene systems was similar to that of 25Pt‐Co@Ti_3_C_2_T*_x_*. For example, using NiCl_2_, PtCl_2_ and Ti_3_AlC_2_ as reactants, Pt‐Ni@Ti_3_C_2_T*_x_* could be obtained. Likewise, when NiCl_2_ was changed to FeCl_2_ or CuCl_2_, and reacting with PtCl_2_ and Ti_3_AlC_2_, the Pt‐Fe@Ti_3_C_2_T*_x_* and Pt‐Cu@Ti_3_C_2_T*_x_* compounds could be prepared. If two or three CoCl_2 ∙6H2O_ , NiCl_2_, FeCl_2_, or CuCl_2_ etching agents were used, Ti_3_C_2_T*_x_*‐supported ternary or quaternary platinum alloys such as Pt‐Ni‐Co@Ti_3_C_2_T*_x_* and Pt‐Fe‐Co‐Ni@Ti_3_C_2_T*_x_* could be prepared. For example, NiCl_2_, CoCl_2_∙6H2O and PtCl_2_ molten salts reacted with Ti_3_AlC_2_ to form Pt‐Ni‐Co@Ti_3_C_2_T*_x_*. In addition, Pt‐Fe‐Co‐Ni@Ti_3_C_2_T*_x_* could be prepared by the reaction of NiCl_2_, CoCl_2∙6H2O_ , FeCl_2_ and PtCl_2_ molten salts with Ti_3_AlC_2_. Al‐containing MAX could be extended to Ti_2_AlC and Ti_3_AlCN to produce Pt‐Co@Ti_3_CNT*_x_* and Pt‐Co@Ti_2_CT*_x_* catalysts. Specific synthesis conditions are shown in Table S1 (Supporting Information).

### Characterization

The morphology and chemical composition of the as‐prepared samples were investigated using XRD, Bruker D8 ADVANCE X‐ray diffractometer with Cu Kα radiation), a scanning electron microscope (Merlin compact, Carl Zeiss AG, Germany) at 5 kV, equipped with an energy dispersive X‐ray spectrometer (Oxford X‐Max) at 20 kV, and TEM(JEM‐2100). The STEM images were acquired using a CEOS probe‐corrected JEOL JEM‐ARM300 TEM at an electron accelerating voltage of 300 kV with a probe convergence angle of 17.8 mrad, spatial resolution of 0.08 nm, and probe current of ≈20 pA. The inner semi‐angular angle of the high‐angle annular dark field (HAADF) detector was 45 mrad. The HAADF‐STEM images were filtered using a standard high‐pass filtering method to reduce noise. XPS was performed using a VG Thermo ESCALAB 250 spectrometer with an excitation source. The binding energy scale was calibrated by adjusting the C 1s peak to 284.8 eV. Peak fitting was performed using Casa XPS. Raman spectroscopy was performed using a Lab RAMHR800 instrument (Jobin Yvon, France) equipped with an air‐cooled CCD array detector in a backscattering configuration. Co‐K‐edge and Pt‐L_3_‐edge XAS data were collected at the BL14W1 beamline at the Shanghai Synchrotron Radiation Facility (SSRF) with a Si (111) double‐crystal monochromator and analyzed using the standard procedures in Demeter^[^ ^41^ ^]^ and HAMA.^[^ ^42^ ^]^ Energy calibration was performed using Co (7709 eV) and P (11564 eV) foils. The Pt content was determined using inductively coupled plasma optical emission spectroscopy (ICP‐OES, Spectro Arcos)

### Electrochemical Measurements

Catalytic performance tests were conducted with a three‐electrode system using an AutoLab electrochemical workstation (PGSTAT 302 N, Metrohm Auto Lab Co., Ltd.). Eight mg of catalyst, 4 mg of acetylene black, 840 µL of ethanol, and 160 µL of Nafion solution (5 wt.%) were thoroughly mixed and ultrasonicated to form a uniform suspension. Five µL of the suspension was dropped onto a rotating disk electrode (RDE) (diameter: 5 mm, mass loading ≈0.2 mg cm^−2^), dried on a clean bench in air for several hours, and served as the working electrode. A Hg/HgO (1.0 M KOH) electrode and graphite rod were employed as the reference and counter electrodes. LSV polarization curves were measured in 1.0 M KOH aqueous electrolyte solution using the RDE at 1600 rpm with a scan rate of 10 mV s^−1^. EIS was performed over a frequency range of 100 kHz to 100 mHz at a current density of 10 mA cm^−2^. The potentials were calibrated with respect to the reversible hydrogen electrode as follows: E_RHE_ = E_Hg/HgO_ + 0.0591×pH + 0.098.

### Computational Details

DFT calculations were performed using the Vienna ab initio Simulation Package (VASP)^[^ ^43^, ^44^ ^]^ in conjunction with the projector augmented wave formalism.^[^ ^45^ ^]^ The H 1s^1^, C 2s^2^2p^2^, O 2s^2^2p^4^, Ti 4s^2^3d^2^, Co 4s^2^3d^7^, and Pt 6s^1^5d^9^states were treated as valence electrons. The electronic wave functions were expanded as plane waves using an energy cut‐off of 500 eV, and the force and energy convergence criteria were set to 0.02 eV Å^−1^ and 10^−5^ eV, respectively. To model the structure of Pt‐Co@Ti_3_C_2_T*_x_*, ‐O‐terminated Ti_3_C_2_T*_x_* was used as the substrate, and the Pt‐Co alloys were exposed at the (111) catalytic surface. The Pt‐Co alloys were based on a Pt alloy with a 1:1 atomic ratio of Pt to Ni, according to the experimental results. The slab model of Pt‐Co@Ti_3_C_2_T*_x_* contained five atomic layers of Ti_3_C_2_T*_x_* and three atomic layers of Pt‐Co alloy. To model the surface reaction, a Monkhorst‐Pack *k*‐point mesh^[^ ^46^ ^]^ of 5×5×1 was used for structural optimization and static self‐consistency. To prevent spurious interactions, a vacuum spacing of ≈15 Å was applied in the z‐direction. ΔG_H_ was considered the key parameter to describe the HER activity.^[^ ^47^ ^]^ The HER in an acidic solution is a two‐step process that involves only one reaction intermediate, the chemisorbed H atom. The free energy of the adsorbed hydrogen is defined as ΔG_H_ = ΔE_H_ + ΔE_ZPE_ ‐TΔS_H_, where ΔE_H_ is the hydrogen adsorption energy, and ΔE_ZPE_ and ΔS_H_ represent the zero‐point energy and entropy correction of the absorbates on the surface.

## Conflict of Interest

The authors declare no conflict of interest.

## Author Contributions

Y.W. and L.L. contributed equally to this work. The manuscript was written through the contributions of all authors. All authors have given approval to the final version of the manuscript.

## Supporting information

Supporting Information

## Data Availability

The data that support the findings of this study are available on request from the corresponding author. The data are not publicly available due to privacy or ethical restrictions.
