# Supplementary material for: Simple One‐Step Molten Salt Method for Synthesizing Highly Efficient MXene‐Supported Pt Nanoalloy Electrocatalysts
Source: Adv Sci (Weinh). 2023 Oct 20;10(33):2303693. doi: 10.1002/advs.202303693 (PMC10667796; doi:10.1002/advs.202303693)
Supplement: Supplementary file 1 — Supporting Information [file ADVS-10-2303693-s001.pdf]

ADVANCED SCIENCE
Open Access

Supporting Information

Simple One-step Molten Salt Method to Synthesize Highly Efficient MXene-supported Pt Nanoalloy Electrocatalysts

Ya Wang[a]‖, *Lili Li*[b]‖, *Miao Shen*[a,c*], *Rui Tang*[a,c*], *Jing Zhou*[a,c], *Ling Han*[a,c], *Xiuqing Zhang*[d], *Linjuan Zhang*[a,c], *Guntae Kim*[a,c], *Jian-Qiang Wang*[a,c*]

Y. Wang, R. Tang, J. Zhou, L. Han, L. Zhang, G. Kim, J. Q. Wang

Shanghai Institute of Applied Physics, Chinese Academy of Sciences, Shanghai201800, China, University of Chinese Academy of Sciences, Beijing 100049, China

L. Li

State Key Laboratory of Crystal Materials and Institute of Crystal Materials, Shandong University, Jinan 250100, China

X. Zhang

East China University of Science and Technology, Shanghai 200237, China

E-mail: shenmiao@sinap.ac.cn, tangrui@sinap.ac.cn, wangjianqiang@sinap.ac.cn

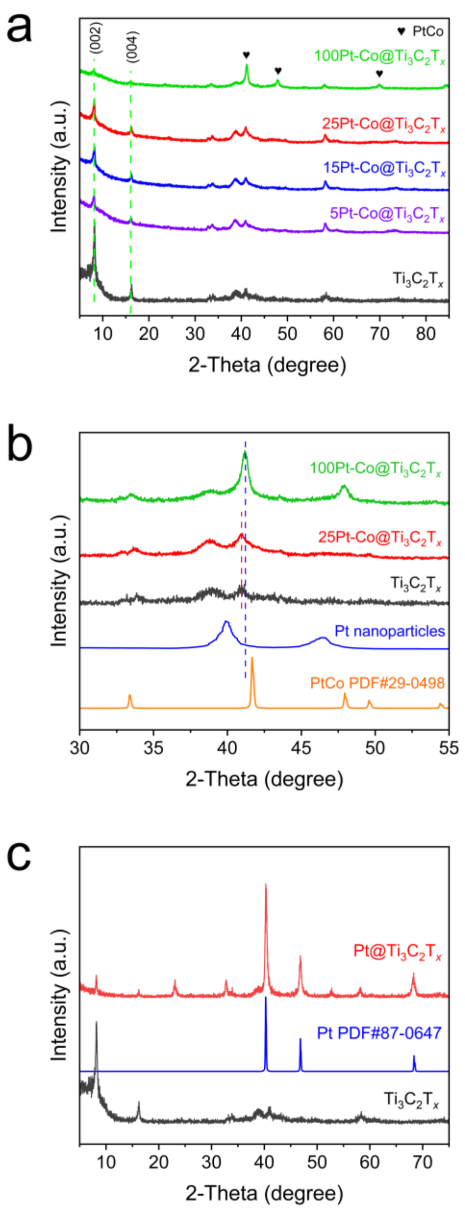

Figure S1. X-ray diffraction (XRD) patterns of as-synthesized (a) 5Pt-Co@$Ti_3C_2T_x$, 15Pt-Co@$Ti_3C_2T_x$, 25Pt-Co@$Ti_3C_2T_x$, and $Ti_3C_2T_x$, (b)100Pt-Co@$Ti_3C_2T_x$, and (c) Pt-Co@$Ti_3C_2T_x$.

The Pt@$Ti_3C_2T_x$ sample was prepared by the reaction of $PtCl_2$ and Ti_3AlC_2 in the molten salt, which resulted in a shift of the (002) peak from 9.86° to 8.11°, indicating a conversion from pristine Ti_3AlC_2 to $Ti_3C_2T_x$. Meanwhile, new peaks at 40.3°, 46.8° and 68.4° appeared, which are corresponding to the (111), (200), and (220) peaks of pure Pt （Pt PDF#87-0647）.

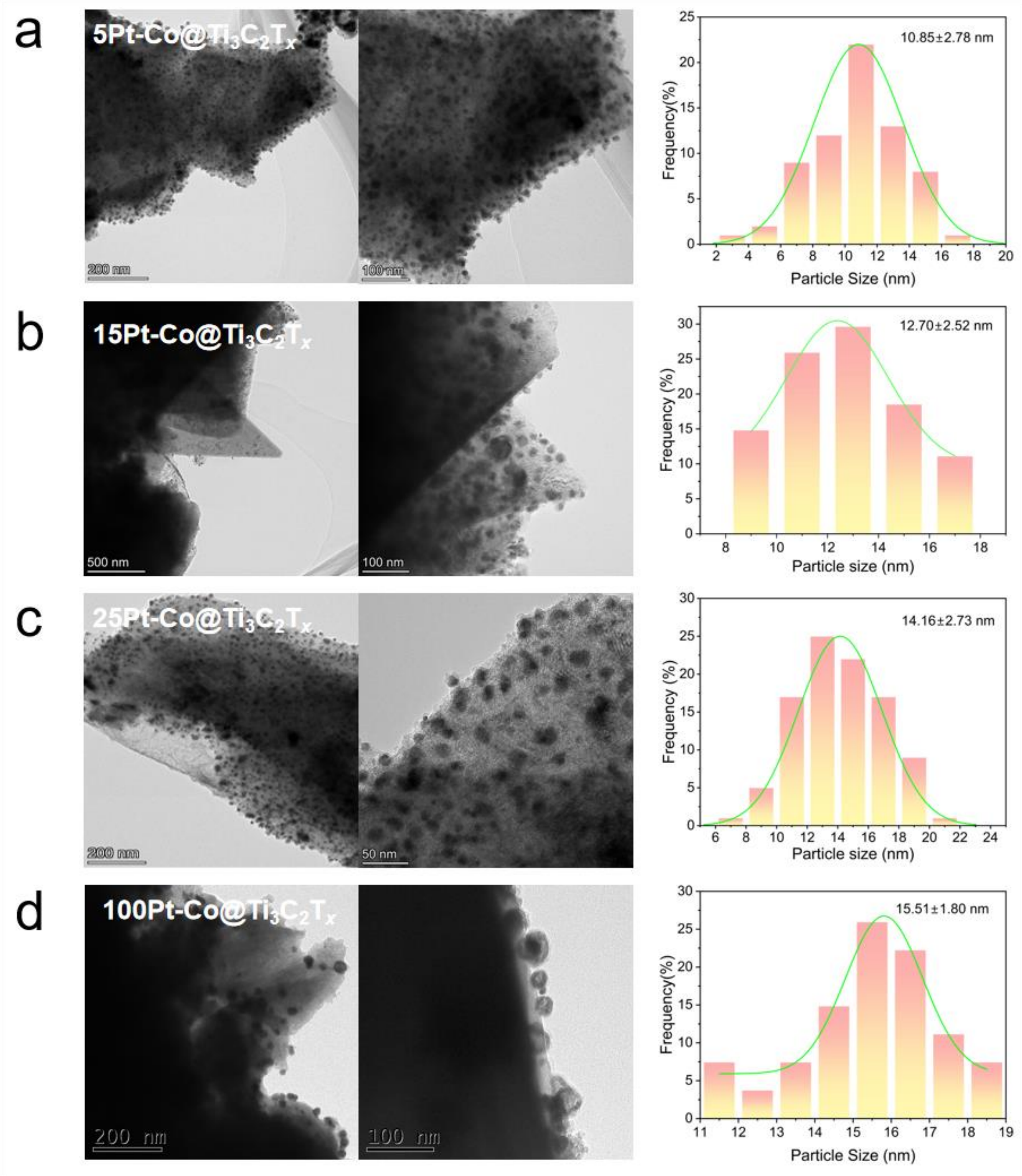

Figure S2. TEM images and sphere size distribution of (a) 5Pt-Co@$Ti_3C_2T_x$, (b)15Pt-Co@$Ti_3C_2T_x$, (c) 25Pt-Co@$Ti_3C_2T_x$ and (d) 100Pt-Co@$Ti_3C_2T_x$.

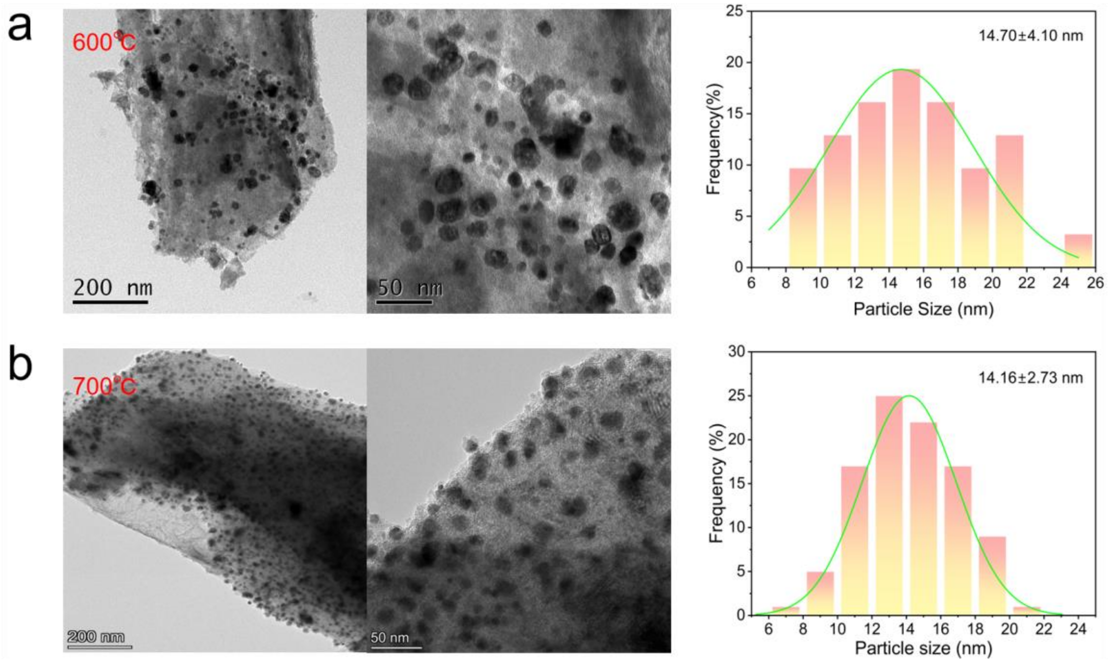

Figure S3. TEM images and sphere size distribution of 25Pt-Co@$Ti_3C_2T_x$ obtained at (a) 600 ℃ and (b) 700 ℃.

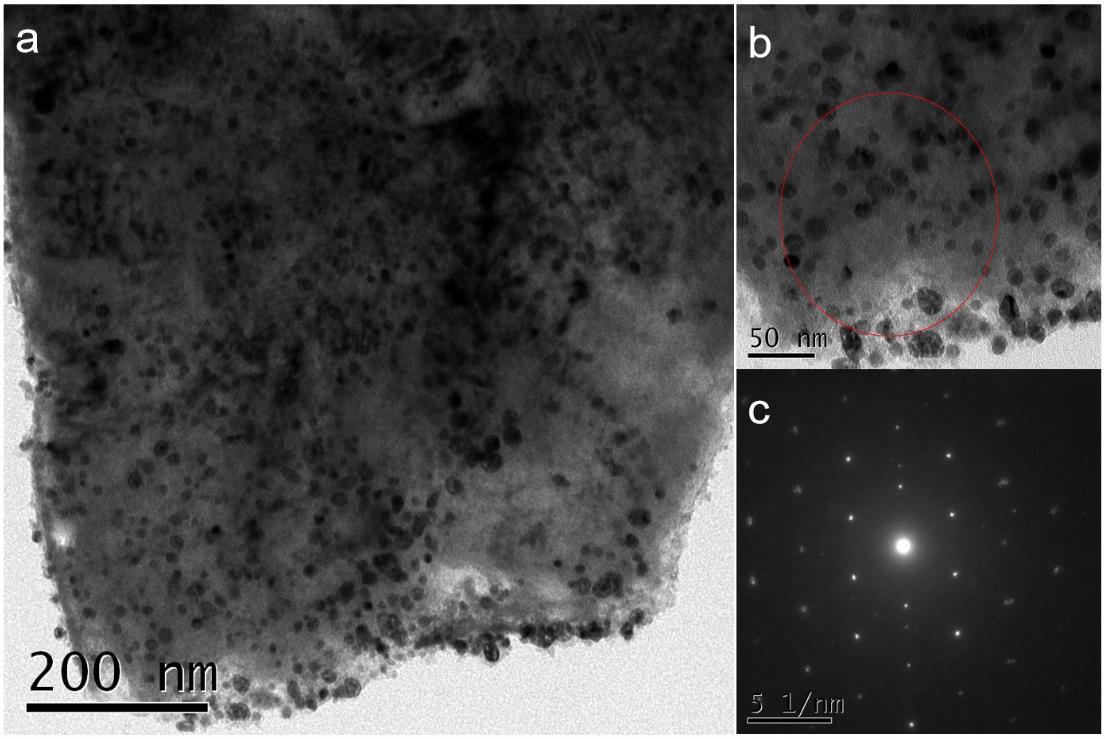

Figure S4. (a) (b) TEM images and (c) SAED pattern of 25Pt-Co@$Ti_3C_2T_x$.

The selected-area electron diffraction (SAED) pattern shows a regular hexagonal lattice, which is in good agreement with the lattice pattern of $Ti_3C_2T_x$.

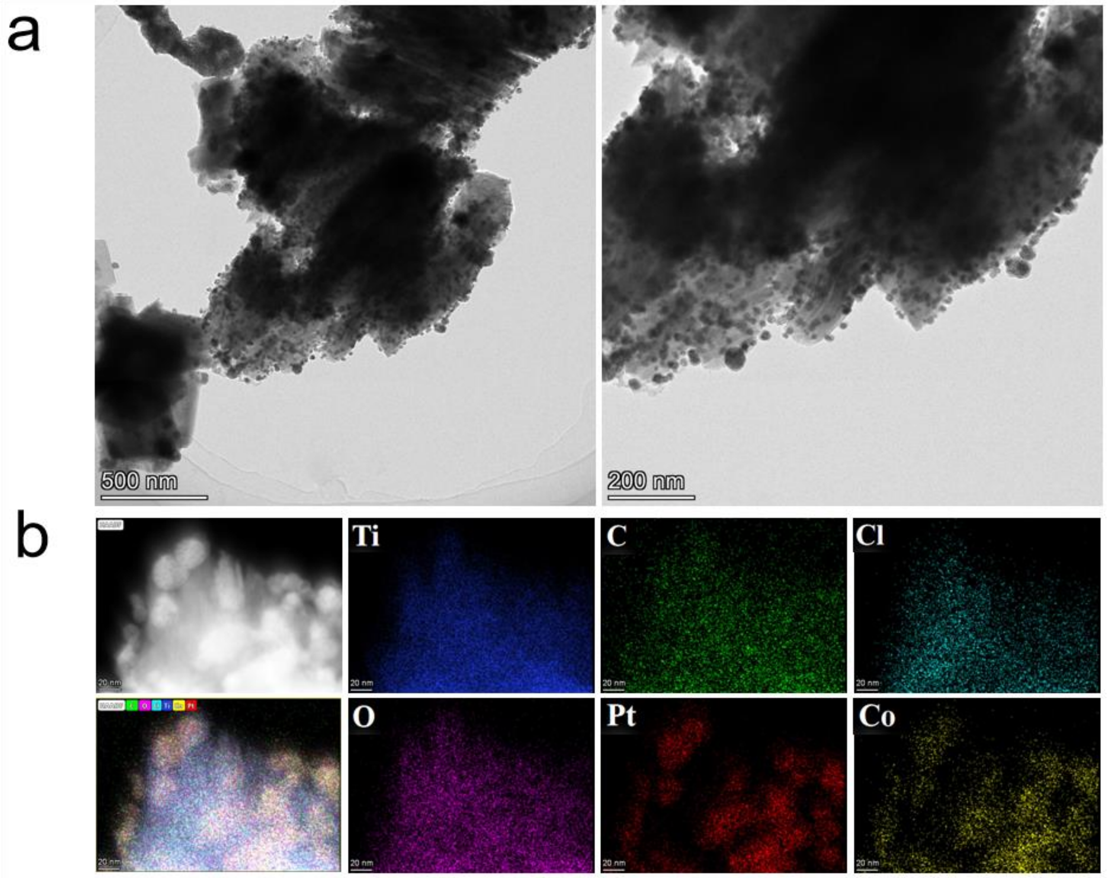

Figure S5. (a)TEM images and (b)the related EDS mapping of Pt, Co, O, Cl, C and Ti elements for 25Pt-Co@$Ti_3C_2T_x$.

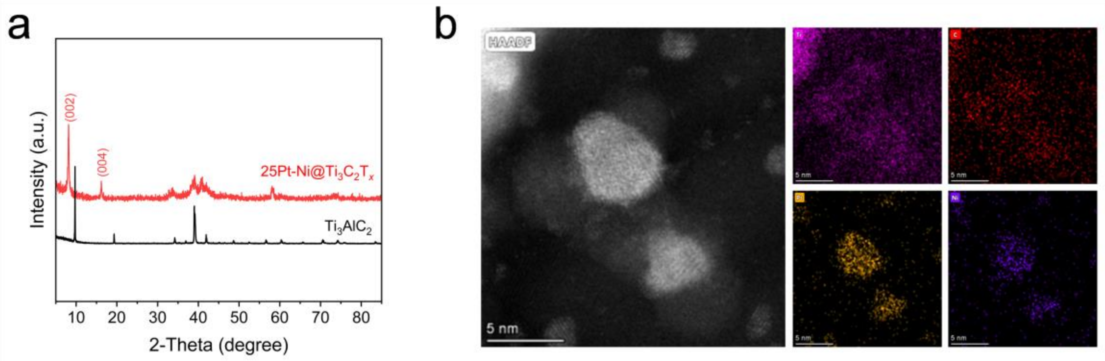

Figure S6. (a) XRD patterns of Ti_3AlC_2 and 25Pt-Ni@$Ti_3C_2T_x$, and (b) HAADF-STEM of 25Pt-Ni@$Ti_3C_2T_x$.

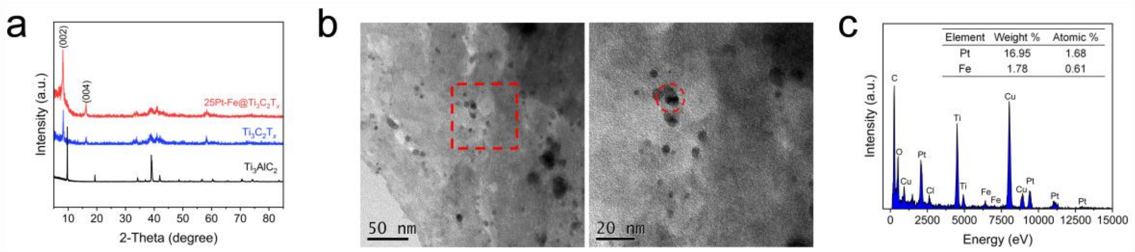

Figure S7. (a) XRD patterns of Ti_3AlC_2, $Ti_3C_2T_x$ and 25Pt-Fe@$Ti_3C_2T_x$, (b)TEM mages and (c) EDX of 25Pt-Fe@$Ti_3C_2T_x$ materials.

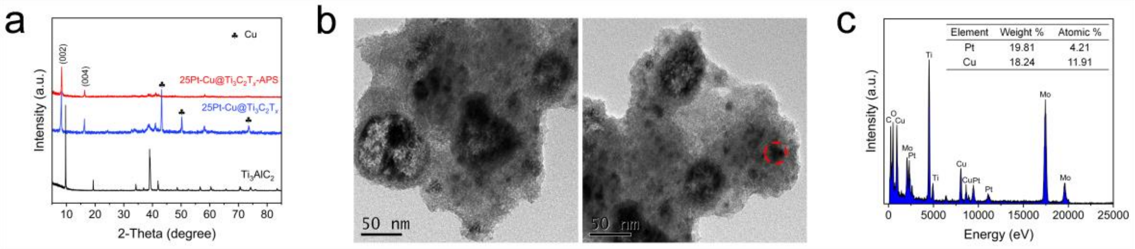

Figure S8. (a) XRD patterns of Ti_3AlC_2 and 25Pt-Cu@$Ti_3C_2T_x$ washed with deionized water and ammonium persulfate, (b) TEM mages and (c) EDX of 25Pt-Cu@$Ti_3C_2T_x$ materials.

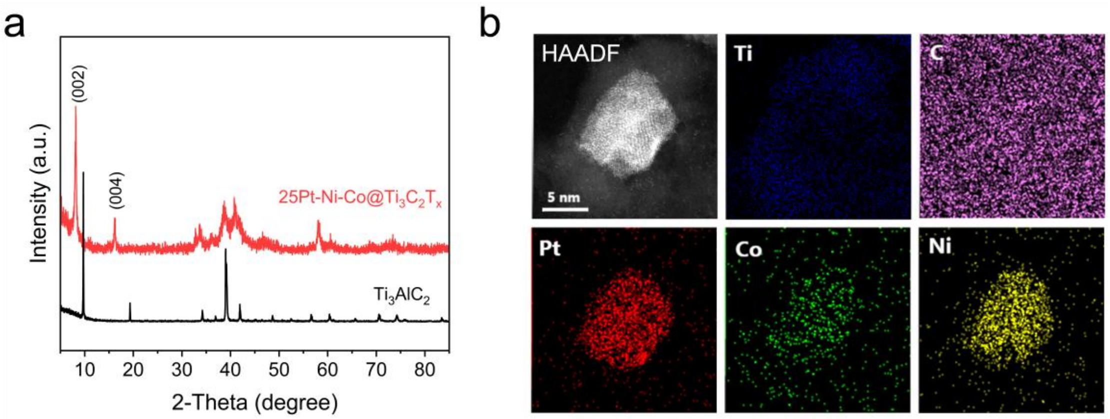

Figure S9. (a) XRD patterns of Ti_3AlC_2, and 25Pt-Ni-Co@$Ti_3C_2T_x$, and (b) HAADF-STEM of 25Pt-Ni-Co@$Ti_3C_2T_x$ materials.

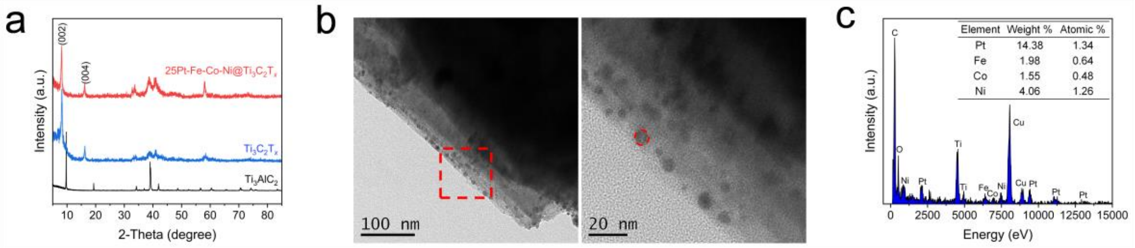

Figure S10. (a) XRD patterns, (b) TEM mages and (c) EDX data of 25Pt-Fe-Co-Ni@$Ti_3C_2T_x$ materials.

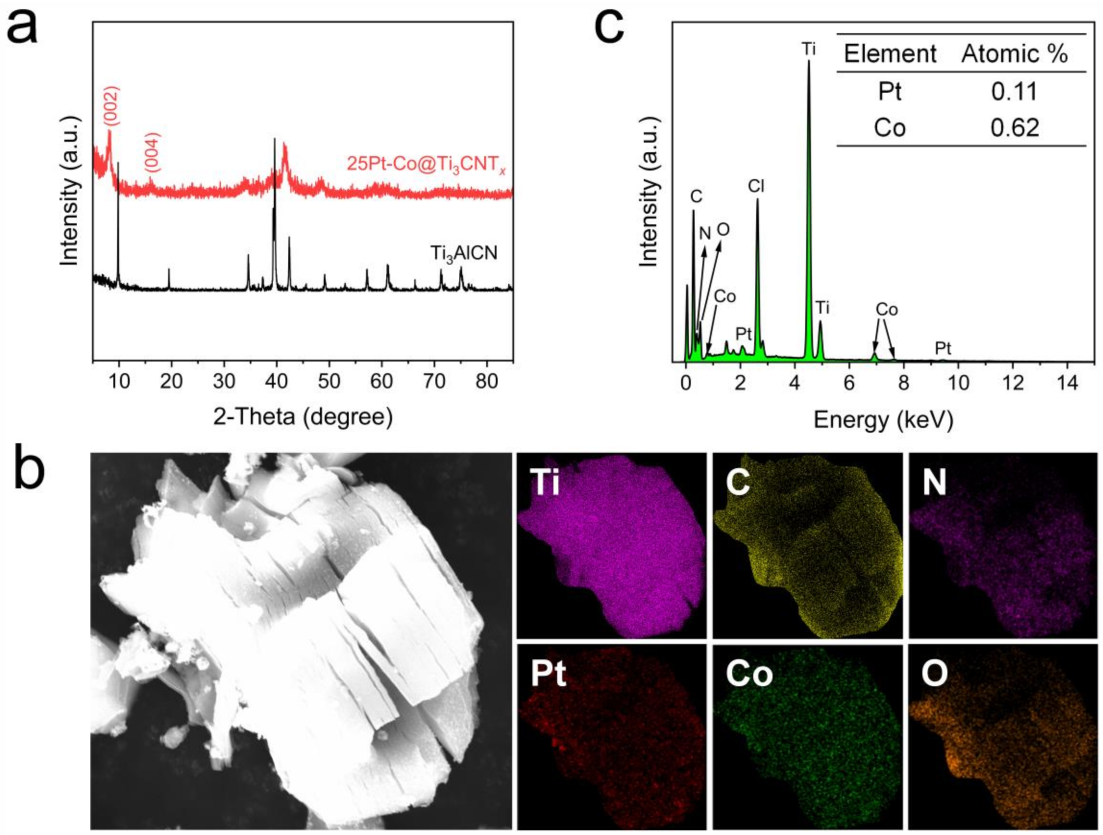

Figure S11. (a) XRD pattern, b) SEM mage and (c) EDS data of 25Pt-Co@Ti_3CNT_x materials.

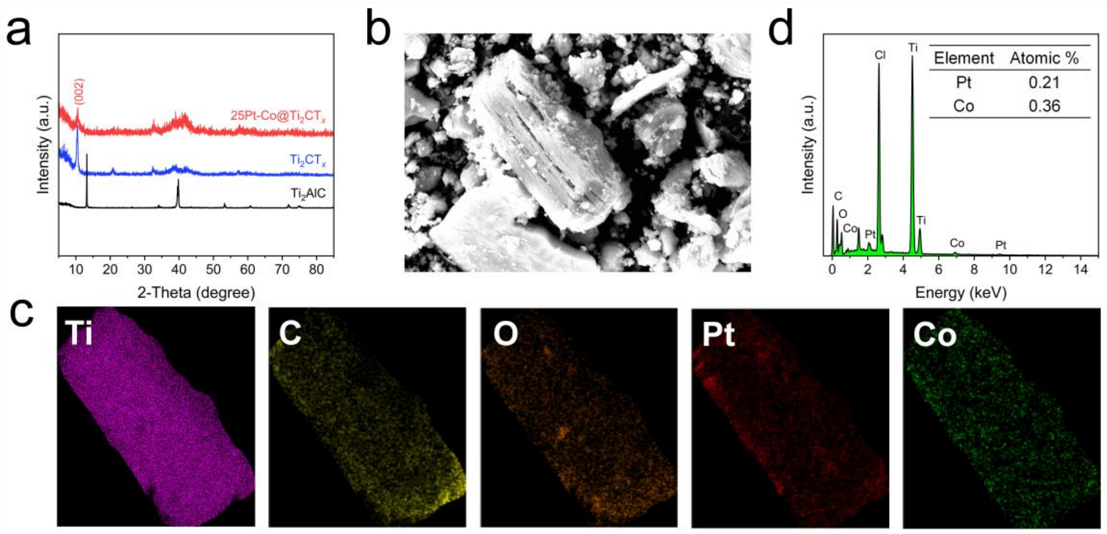

Figure S12. (a) XRD pattern, (b) SEM mage, (c) EDS data and (d) EDS mapping of 25Pt-Co@Ti_2CT_x materials.

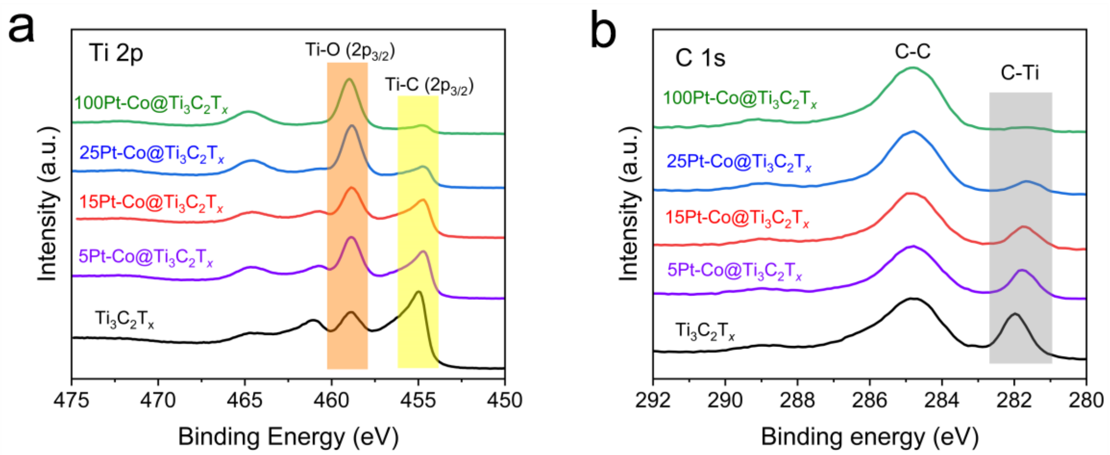

Figure S13. High-resolution XPS spectra of (a) Ti 2p and (b) C 1s of $Ti_3C_2T_x$ and Pt-Co@$Ti_3C_2T_x$ with different concentration of Pt.

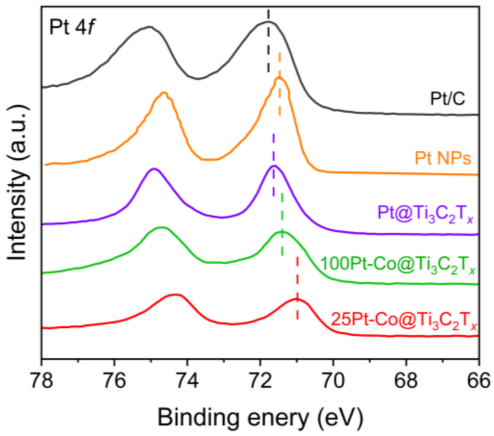

Figure S14. High-resolution XPS spectra of Pt 4f.

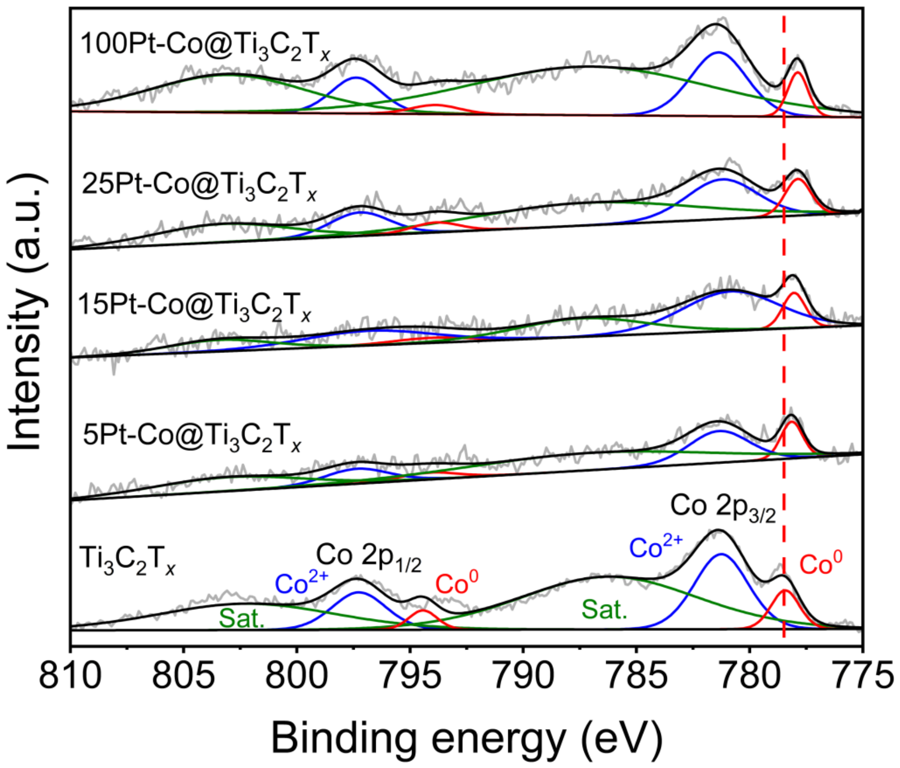

Figure S15. High-resolution XPS spectra of Co 2p of $Ti_3C_2T_x$ and Pt-Co@$Ti_3C_2T_x$ with different concentration of Pt.

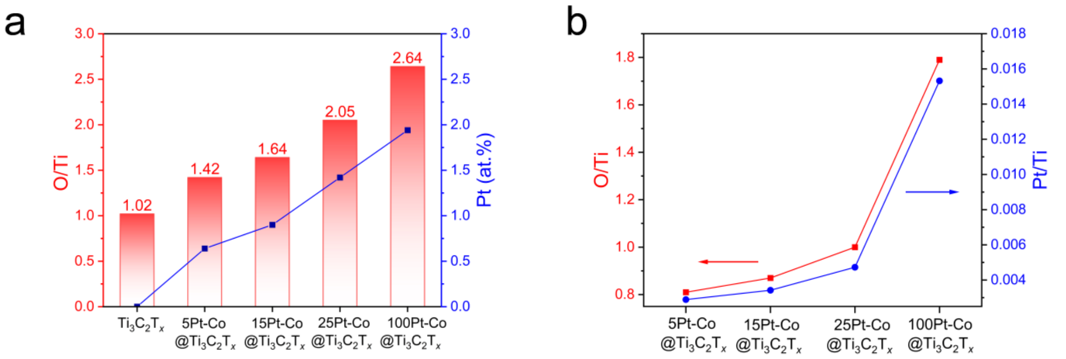

Figure S16. The ratio of O to Ti in Pt-Co@$Ti_3C_2T_x$ with different concentration of Pt.(a) determined from XPS.(b)determined from EDS.

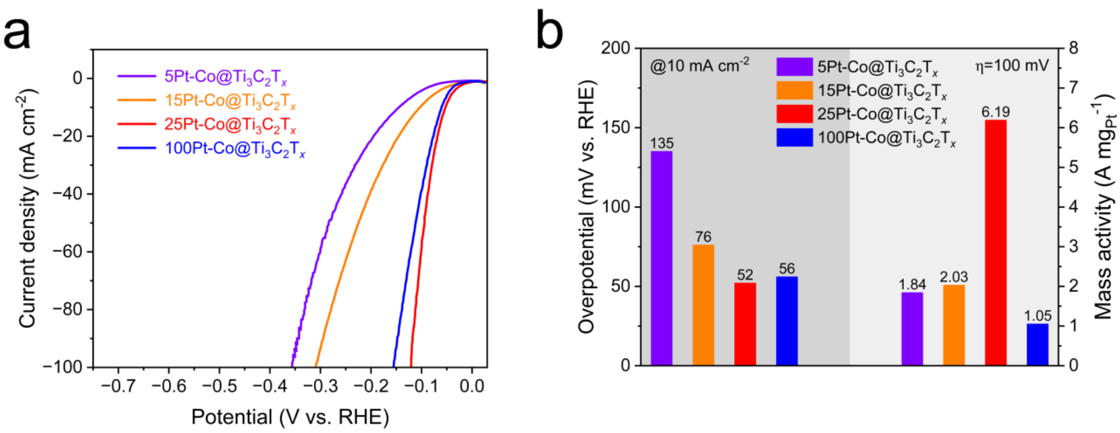

Figure S17. (a) LSV polarization curves of Pt-Co@$Ti_3C_2T_x$ with different concentration of Pt and (b) Specific activity of electrocatalysts at 10 mA cm^{-2} and overpotential of 100 mV.

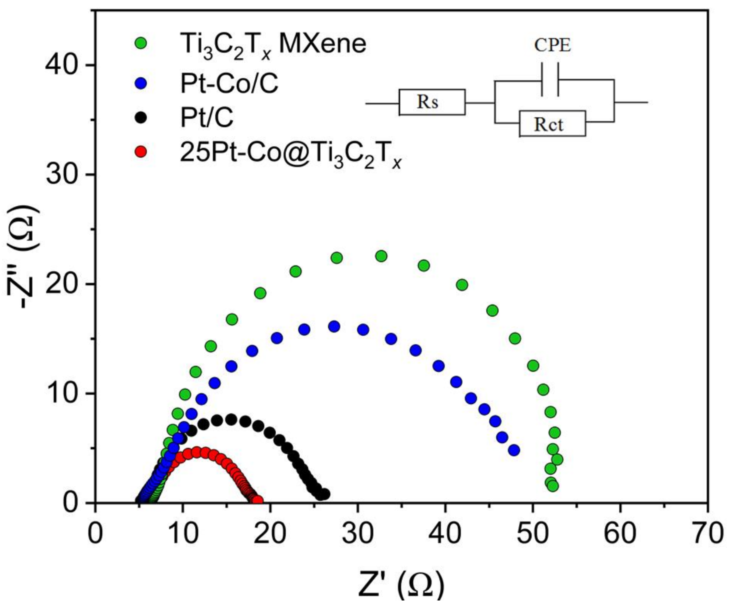

Figure S18. Nyquist plots measured at -10 mA cm^{-2}.

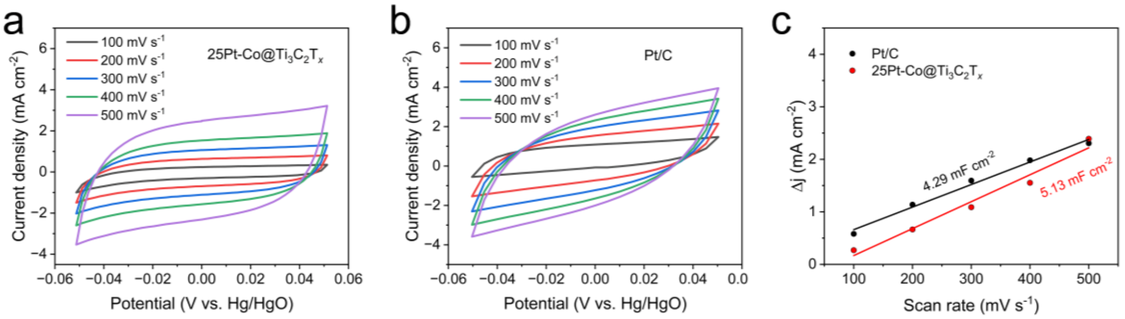

Figure S19. CV curves at different scan rates (100, 200, 300, 400, and 500 mV·s^{-1}) in 1 M KOH solution for (a) 25Pt-Co@$Ti_3C_2T_x$, (b) commercial Pt/C. (c) Current density variation plotted against scan rates of two samples.

The CV curves in non-faradaic region and corresponding current density variation against scan rates plot are shown in Figure S19. The 25Pt-Co@$Ti_3C_2T_x$ exhibits the slope of 5.13 mF cm^{-2}, larger than that of commercial Pt/C (4.29 mF cm^{-2}), indicating an improved ECSA for 25Pt-Co@$Ti_3C_2T_x$ in alkaline solution.

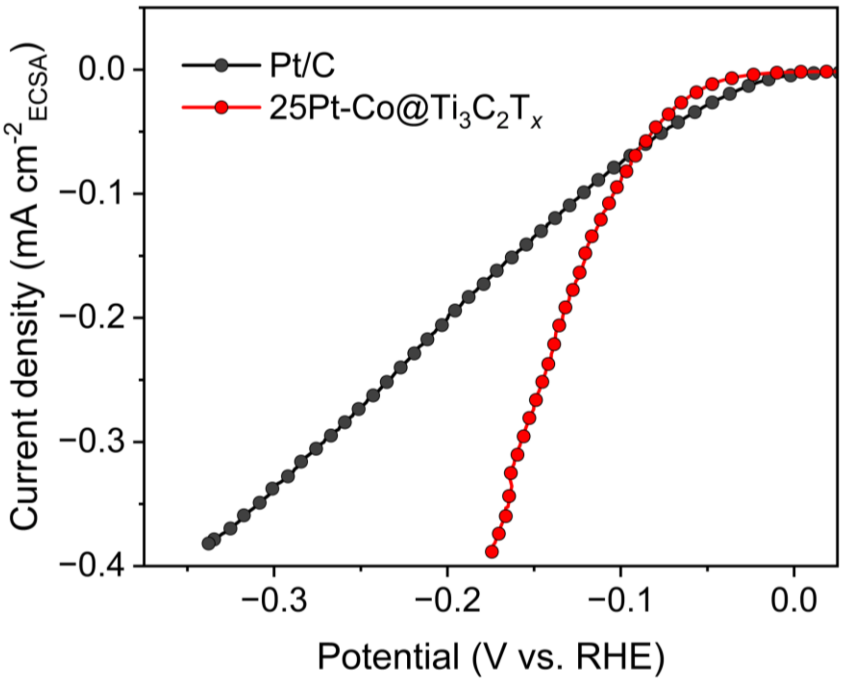

Figure S20. Electrocatalytic HER performance of 25Pt-Co@$Ti_3C_2T_x$ and commercial Pt/C in 1.0 M KOH electrolyte, specific activity based on ECSA.

The ECSA normalized hydrogen evolution current (j) was used to highlight the intrinsic catalytic activity (Figure S19). The results show that the intrinsic activity of 25Pt-Co@$Ti_3C_2T_x$ is significantly superior to that of Pt/C at higher current densities.

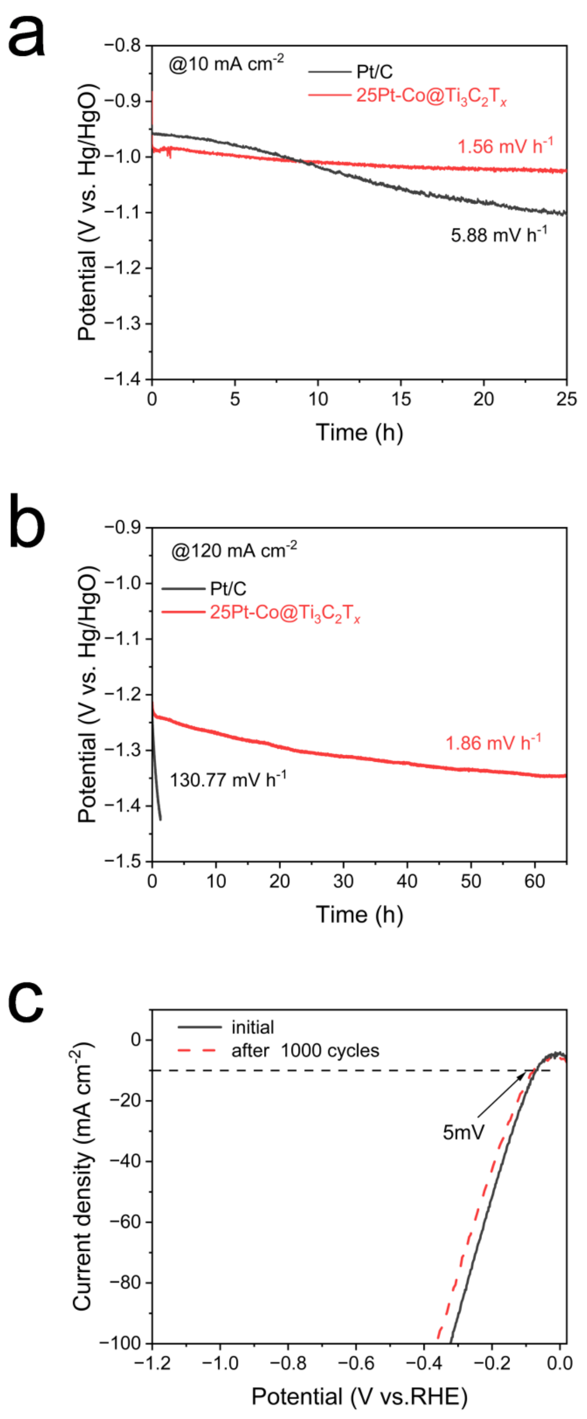

Figure S21. Long-term electrochemical stability of 25Pt-Co@$Ti_3C_2T_x$ and 20wt% Pt/C(a) at -10 mAcm^{-2}, (b) -120 mA cm^{-2} and (c) Polarization curves of 25Pt-Co@$Ti_3C_2T_x$at initial and after 1000 CV cycles.

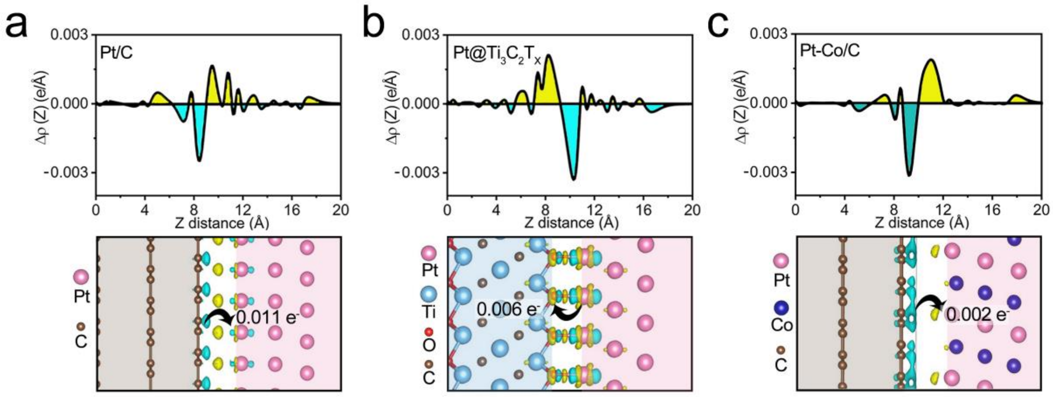

Figure S22. The planar average electron density difference (Δρ) is integrated over the x-y plane for the **a** Pt/C, **b** Pt@$Ti_3C_2T_x$ and **c** Pt-Co/C as a function of the z distance. The shadow is the charge density difference upon the interface. The yellow and cyan colors indicate the electron accumulation and depletion, respectively. The arrow shows the direction of the electron transfer.

Table S1. The synthesis conditions of Pt-M@MXene samples.

Samples	Reactants		Temperature (℃)	Time (h)
$Ti_3C_2T_x$	Ti_3AlC_2 (0.2 g)+$CoCl_2{\cdot}6H_2O$ (0.49 g)	LiCl-KCl (0.24 g)	700	24
5Pt-Co@$Ti_3C_2T_x$	Ti_3AlC_2 (0.2 g)+$CoCl_2 6H_2O$ (0.49 g)+$PtCl_2$ (5 mg)	LiCl-KCl (0.24 g)	700	24
15Pt-Co@$Ti_3C_2T_x$	Ti_3AlC_2 (0.2 g)+$CoCl_2{\cdot}6H_2O$ (0.49 g)+$PtCl_2$ (15 mg)	LiCl-KCl (0.24g)	700	24
25Pt-Co@$Ti_3C_2T_x$	Ti_3AlC_2 (0.2 g)+$CoCl_2{\cdot}6H_2O$ (0.49 g)+$PtCl_2$ (25 mg)	LiCl-KCl (0.24 g)	700	24
100Pt-Co@$Ti_3C_2T_x$	Ti_3AlC_2 (0.2 g)+$CoCl_2{\cdot}6H_2O$ (0.49 g)+$PtCl_2$ (100 mg)	LiCl-KCl (0.24 g)	700	24
25Pt-Co@$Ti_3C_2T_x$	Ti_3AlC_2(0.2g)+$CoCl_2{\cdot}6H_2O$ (0.49g)+$PtCl_2$ (25 mg)	LiCl-KCl (0.24 g)	600	24
25Pt-Fe@$Ti_3C_2T_x$	Ti_3AlC_2(0.2g)+$FeCl_2$ (0.27g)+ $PtCl_2$ (25 mg)	LiCl-KCl (0.24 g)	700	24
25Pt-Ni@$Ti_3C_2T_x$	Ti_3AlC_2 (0.2 g)+ $NiCl_2$ (0.27 g)+$PtCl_2$ (25 mg)	LiCl-KCl (0.24 g)	700	24
25Pt-Cu@$Ti_3C_2T_x$	Ti_3AlC_2 (0.2 g)+ $CuCl_2$ (0.27 g)+$PtCl_2$ (25 mg)	LiCl-KCl (0.24 g)	700	24
25Pt-Ni-Co@$Ti_3C_2T_x$	Ti_3AlC_2 (0.2 g)+$CoCl_2{\cdot}6H_2O$ (0.49 g)+$NiCl_2$ (0.27g)+ $PtCl_2$ (25 mg)	LiCl-KCl (0.24 g)	700	24
25Pt-Fe-Co-Ni@$Ti_3C_2T_x$	Ti_3AlC_2 (0.2 g)+$CoCl_2{\cdot}6H_2O$ (0.49 g)+$FeCl_2$ (0.27 g)+ $NiCl_2$ (0.27 g)+$PtCl_2$ (25 mg)	LiCl-KCl (0.24 g)	700	24
25Pt-Co@Ti_3CNT_x	Ti_3AlCN (0.2 g)+$CoCl_2{\cdot}6H_2O$ (0.49 g)+$PtCl_2$ (25 mg)	LiCl-KCl (0.24 g)	700	24
25Pt-Co@Ti_2CT_x	Ti_2AlC (0.2 g)+$CoCl_2{\cdot}6H_2O$ (0.49 g)+$PtCl_2$ (25 mg)	LiCl-KCl (0.24 g)	700	24
Pt@$Ti_3C_2T_x$	Ti_3AlC_2 (0.037 g)+ $PtCl_2$ (100 mg)	LiCl-KCl (0.24 g)	700	7

Table S2. The content of Pt and Co elements in Pt-Co@$Ti_3C_2T_x$samples with different concentration of Pt determined by ICP-OES.

**Catalysts**	***Pt (wt%)***	***Co (wt%)***	***Pt:Co (atom)***
5Pt-Co@$Ti_3C_2T_x$	1.29	1.55	0.25:1
15Pt-Co@$Ti_3C_2T_x$	2.30	1.41	0.49:1
25Pt-Co@$Ti_3C_2T_x$	4.68	1.58	0.90:1
100Pt-Co@$Ti_3C_2T_x$	18.08	3.91	1.40:1

Table S3. The content of Pt and M elements in 25Pt-M@$Ti_3C_2T_x$ samples determined by ICP-OES.

Catalysts	*Pt (wt%)*	*Co (wt%)*	*Ni (wt%)*	*Fe (wt%)*	*Zn (wt%)*
25Pt-Co@$Ti_3C_2T_x$	4.68	1.58	/	/	/
25Pt-Ni@$Ti_3C_2T_x$	4.34	/	2.79	/	/
25Pt-Fe@$Ti_3C_2T_x$	3.83	/	/	1.41	/
25Pt-Zn@$Ti_3C_2T_x$	2.50	/	/	/	1.58

Table S4. Curve-Fitting Parameters for Ti 2p XPS Spectra of $Ti_3C_2T_x$ and Pt-Co@$Ti_3C_2T_x$ with different concentration of Pt.

**Samples**	***Ti-C***		***Ti with higher valence state***	
	Ti-C (I)	Ti-C (II)	Ti-Cl / Ti^{3+}	Ti-O
$Ti_3C_2T_x$	12.72	20.52	41.05	**25.71**
5Pt-Co@$Ti_3C_2T_x$	15.39	21.3	34.86	**28.45**
15Pt-Co@$Ti_3C_2T_x$	13.08	14.4	29.61	**42.91**
25Pt-Co@$Ti_3C_2T_x$	8.45	12.07	35.31	**44.17**
100Pt-Co@$Ti_3C_2T_x$	3.6	4.08	23.01	**69.30**
Pt@$Ti_3C_2T_x$	13.16	16.59	32.37	**37.88**

Table S5. Curve-Fitting Parameters for C 1s XPS Spectra of $Ti_3C_2T_x$ and Pt-Co@$Ti_3C_2T_x$ with different concentration of Pt.

**Samples**	***C-Ti***	*C-C*	C-O	O-C=O
$Ti_3C_2T_x$	**22.06**	56.79	12.09	8.26
5Pt-Co@$Ti_3C_2T_x$	**18.45**	65.72	7.15	8.68
15Pt-Co@$Ti_3C_2T_x$	**14.95**	66.57	9.7	8.78
25Pt-Co@$Ti_3C_2T_x$	**8.64**	72.21	10.12	9.03
100Pt-Co@$Ti_3C_2T_x$	**2.82**	63.23	24.73	9.20
Pt@$Ti_3C_2T_x$	**15.71**	46.02	28.66	9.61

Table S6. The EIS fitting parameters for all samples.

Samples	R_s / Ω	R_{ct} / Ω
25Pt-Co@$Ti_3C_2T_x$	6.5	10.9
Pt/C	6.2	18.6
Pt-Co/C	6.1	41.5
$Ti_3C_2T_x$	6.4	45.2

Table S7. HER performance of Pt-based catalysts in 1.0 M KOH.

Catalysts	Pt loading (μg cm^{-2})	Particle Size (nm)	Overpotential @10 mAcm^{-2} (mV)	Tafel slope	Mass activity (A mg pt^{-1})	Reference
25Pt-Co@Ti$_3$C$_2$T$_x$	**9.5**	**14**	**52**	**52.6**	**6.19** (100 mV)	**This work**
MoC$_{1-x}$/Pt-600-NPs	54	18.2	67	55	~0.22 (70 mV)	1
Pt@NiFe LDHs	41.1	4	101	127	~0.19 (70 mV) ~0.243(100mV)	2
Pt-Co(OH)$_2$/CC	390	3.5	84	70	0.004 (70 mV)	3
Ni-MOF@Pt	40	3.23	102	52	0.241(100 mV)	5
Ni$_3$N/Pt	~300	10	50	36.5	~0.08 (70 mV)	6
β-Ni(OH)$_2$/Pt	13	/	108	39	/	7
Pt NWs/SL-Ni(OH)$_2$	16.1	~2	70	/	0.68 (70 mV)	8
Pd(100)-Pt	20	2.4	71	31	~0.37(Pt+Pd) (70mV)	9
Pt@PCM	107.1	14	139	73.6	1.6 (500mV)	10
Pt SA-PNPM	9.5	single-atom	36	33	4.18 (75mV)	11
2.4%Pt@mh-3D MXene	4.8	1-2	27	41	12.94 (100 mV)	12
PtNi@Ti$_3$C$_2$	3.4	5	36	59	6.31 (70 mV)	13
20 % Pt/Ni(HCO$_3$)$_2$	40	2.36	27	45	1.77 (100 mV)	14
Pt-Ni/NiS NWs	15.3	~20	51	/	2.28 (100 mV)	15
Pt$_3$Ni$_3$-NWs	15.3	~20	40	/	4.58 (100 mV)	16
Pt$_{3.6}$Ni-S	7.6	~2.6	20	114.7	6.04(100 mV)	17

References

[1] H. J.Song, M.-C. Sung, H. Yoon, B.Ju, D.-W.Kim,*Adv. Sci.* **2019**, *6*, 1802135.

[2] S. Anantharaj, K. Karthick, M. Venkatesh,T.V.S. V.Simha, A. S.Salunke, L.Ma, H. Liang, S. Kundu, *Nano Energy* **2017**, *39*, 30.

[3] Z.Xing,C.Han, D.Wang, Q.Li, X. Yang, *ACS Catal.* **2017**, *7*, 7131.

[4] K.Jiang, B.Liu, M.Luo, S.Ning, M.Peng,Y.Zhao, Y.-R.Lu, T.-S.Chan, F. M. F.de Groot, Y. Tan, *Nat. Commun.* **2019**, *10*, 1743.

[5] K.Rui,G.Zhao, M.Lao, P.Cui, X.Zheng, X.Zheng, J.Zhu, W.Huang, S. X.Dou, W.Sun, *Nano Lett.* **2019**, *19*, 8447.

[6] Y.Wang, L.Chen,X. Yu, Y. Wang, G.Zheng, *Adv. Energy Mater.***2017**, *7*, 1601390.

[7] X.Yu, J.Zhao,L.-R. Zheng, Y.Tong,M. Zhang,G. Xu, C.Li,J. Ma,G. Shi, *ACS Energy Lett.* **2018**,*3*, 237.

[8] H.Yin,S. Zhao,K. Zhao, A. Muqsit, H. Tang, L. Chang, H. Zhao, Y. Gao, Z. Tang,*Nat. Commun.* **2015**, *6* , 6430.

[9] J.Fan, K. Qi, L. Zhang, H. Zhang, S.Yu, X.Cui,*ACS Appl. Mater. Interfaces* **2017**, *9*, 18008.

[10] H.Zhang, P. An,W. Zhou, B. Y. Guan, P.Zhang, J. Dong, X. W. D. Lou, *Sci. Adv.* **2018**, *4*, eaao6657.

[11] W. Peng, J. Han, Y. Lu, M. Luo,T. Chan, M. Peng,Y. Tan, *ACS Nano* **2022**, *16*, 4116.

[12] L. Xiu, W. Pei, S. Zhou, Z. Wang, P. Yang, J. Zhao, J. Qiu, *Adv. Funct. Mater.* 2020, *30*, 1910028.

[13] Y.Yan, R.Zhang, Y.Yu, Z.Sun, R. Che, B.Wei, A. P.LaGrow, Z.Wang,W.Zhou, Appl. Catal. B 2021, 291, 120100.

[14] M. Lao, K. Rui, G. Zhao, P. Cui, X. Zheng,S. Dou, W. Sun, *Angew. Chem. Int. Ed.*, **2019**, *58*, 5432.

[15] P.Wang, X. Zhang, J. Zhang,S.Wan, S. Guo.G. Lu, J. Yao, X. Huang, *Nature Commun.*, **2017**, *8*,14580.

[16] P. Wang, K. Jiang, G. Wang, J. Yao, X. Huang, *Angew. Chem. Int. Ed.*, **2016**, *55*, 128,1.

[17] Z. Liu, J. Qi,M. Liu, S. Zhang, Q. Fan, H. Liu, K. Liu,H. Zheng,Y.Yin, C. Gao. *Angew. Chem. Int. Ed.*, **2018**, *57*, 11678.
